# Polishing Mechanism of CMP 4H-SiC Crystal Substrate (0001) Si Surface Based on an Alumina (*Al*_2_*O*_3_) Abrasive

**DOI:** 10.3390/ma17030679

**Published:** 2024-01-31

**Authors:** Juntao Gong, Weilei Wang, Weili Liu, Zhitang Song

**Affiliations:** 1 National Key Laboratory of Materials for Integrated Circuits, Shanghai Institute of Microsystem and Information Technology, Chinese Academy of Sciences, 865 Changning Road, Shanghai 200050, China; ztsong@mail.sim.ac.cn; 2 University of Chinese Academy of Sciences, No. 19, Yuquan Road, Shijingshan District, Beijing 100049, China; 3 Shanghai Xinanna Electronic Technology Co., Ltd., Shanghai 201506, China; awelly@mail.sim.ac.cn; 4 Zhejiang Xinchuangna Electronic Technology Co., Ltd., Haining 314406, China

**Keywords:** SiC, CMP, alumina, polishing mechanism

## Abstract

Silicon carbide, a third-generation semiconductor material, is widely used in the creation of high-power devices. In this article, we systematically study the influence of three crucial parameters on the polishing rate of a silicon carbide surface using orthogonal experiments. By optimizing the parameters of chemical mechanical polishing (CMP) through experiments, we determined that the material removal rate (MRR) is 1.2 μm/h and the surface roughness (Ra) is 0.093 nm. Analysis of the relevant polishing mechanism revealed that manganese dioxide formed during the polishing process. Finally, due to the electrostatic effect of the two, $MnO_{2}$ adsorbed on the $Al_{2}O_{3}$ , which explains the polishing mechanism of $Al_{2}O_{3}$ in the slurry.

## 1. Introduction

Compared to first-generation semiconductor materials (such as Si), third-generation wide-bandgap-power semiconductors exhibit unparalleled advantages such as high thermal conductivity, high electron mobility, wide bandgap, and strong chemical inertness [1]. Currently, SiC is the most well-developed third-generation semiconductor material and has the best comprehensive performance. Compared with silicon, SiC reveals the following advantages: (1) its dielectric breakdown field strength is 10 times that of silicon, which can reduce power loss; (2) its electron saturation speed is twice that of silicon, allowing the device to achieve a faster switching speed; (3) its bandgap width is three times that of Si, reducing the loss of high-power devices; (4) its thermal conductivity is four times that of Si, which has high temperature resistance and improves the integration and power density of the device. These factors support SiC’s widespread application in semiconductor lighting, aerospace and automotive electronic equipment [2,3,4].

The surface quality of a substrate material will directly affect the performance of the device. With the continuous development of semiconductor devices, in pursuit of higher integration and better performance, the requirements for the flatness and surface quality of silicon carbide surfaces are becoming increasingly high. Chemical mechanical polishing (CMP) is an effective machining technique used to planarize the surface of silicon carbide and is currently the only way to achieve global polishing [5]. Chemical–mechanical polishing involves maintaining a certain pressure and relative motion between the test piece and the polishing pad in the polishing fluid (which is composed of solid abrasive particles, oxidants, and liquid media). The oxidant in the polishing fluid is chemically reacted with the surface of the test piece under frictional conditions to form a soft layer that is more easily removed by the abrasive particles in the polishing fluid. This technique involves a mixture of chemical corrosion and mechanical polishing [6].

Polishing fluid is a key factor affecting the performance of silicon carbide CMP. It affects both the mechanical removal process and the chemical reaction process of CMP. Colloidal silica is widely used in the field of integrated circuit polishing [7,8,9]. However, due to the low hardness of silica, the material removal rate and efficiency of polishing SiC substrates with silica abrasive are low. $Al_{2}O_{3}$ is a well-known abrasive material with high hardness (Mohs hardness 9) that can enhance mechanical removal and improve the MRR of SiC-CMP, making it a popular new polishing abrasive [10]. Su et al. [11] investigated the effect of process conditions on the Si-surface of 6H-SiC in polishing solution using alumina as abrasive and results indicated that rotational speed of the platen and polishing pressure have a greater impact on the MRR, while Chen et al. [12] investigated the effects of alumina and silica abrasives on the polishing performance of SiC when a nanosecond laser was combined with CMP and found that oxide structure is formed on the surface of SiC Si-face after laser modulation and the structure and oxide content affect MRR of CMP. Wang et al. [13] studied the effect of $Al_{2}O_{3}$ and $ZrO_{2}$ mixed abrasives on 4H-SiC CMP under photocatalytic conditions. It was found that the electron transition on the surface of $ZrO_{2}$ particles under UV irradiation promotes the decomposition of $H_{2}O_{2}$ into hydroxyl radicals, which is beneficial for solving the problem of the rate-limiting step of SiC-CMP. However, thus far, we have been unable to find a mechanistic study of CMP SiC substrate based on alumina abrasive in the literature.

In this paper, a series of chemical–mechanical polishing tests were performed on the surface of 4H-SiC (0001) Si by using the orthogonal design of abrasive alumina ( $Al_{2}O_{3}$ ) slurry. The effects of the pH, abrasive content, and oxidant content on the material removal rate were studied. Finally, a CMP slurry based on alumina ( $Al_{2}O_{3}$ ) abrasive was obtained. After that, we conducted polishing experiments on 4H-SiC using the optimized slurry while simultaneously analyzing the removal mechanism of silicon carbide under this slurry using characterization techniques such as scanning electron microscopy (SEM), energy-dispersive X-ray spectroscopy (EDS), laser particle size analyzer (LPSA), and X-ray photoelectron spectroscopy (XPS).

## 2. Materials and Methods

In this experiment, we used a 2-inch 4H-SiC single-crystal wafer (N-type, doping nitrogen, off angle = $4^{\circ }$ , density of micropipe < 1/cm^2^, total defect density < $10^{3}/{\mathrm{cm}}^{2}$ ) manufactured by Hebei Tongguang Crystal Co., Ltd., Baoding, China. The polishing process employed alumina as an abrasive, with a particle size of 500 nm. Hypermanganate agents were used as oxidizing agents in the slurry. The pH of the polishing slurry was carefully adjusted with the nitric acid and potassium hydroxide.

The polishing experiment used the CP-4 type polisher from the Bruker. A polyurethane polishing pad (IC-1000 K-Groove) was selected, and a 4-inch diamond dresser (3M-S60) was used to condition the pad. Before each polishing test, we used a diamond dresser to dress the polishing pad for 10 min, and then used paraffin to bond the sample to the stainless steel polishing head. After polishing, we wiped the paraffin on the surface of the sample with anhydrous alcohol, cleaned it with an ultrasonic cleaning machine for 10 min, and then dried the sample with high-purity nitrogen gas. The weight of SiC wafers was determined using the electronic balance (Mettler Toledo AG 285, accuracy: 0.01 mg) before and after polishing, and the material removal rate of the SiC wafer was calculated. The formula for material removal rate (MRR) is as follows:

(1) $$MRR=\frac{m_{0}-m_{1}}{\rho *S*t}$$

where $m_{0}$ is the mass of silicon carbide before polishing, $m_{1}$ is the mass of silicon carbide after polishing, ρ is the density of 4H-SiC, *S* is the area of the silicon carbide wafer, and *t* is the polishing time. The specific parameters of the polishing experiment are shown in Table 1.

In this experiment, atomic force microscope (AFM) from Park Systems in Suwon, Republic of Korea was employed to observe the surface quality of the SiC wafer after polishing. We used SEM (JSM-7800F) from JEOL Ltd. in Tokyo, Japan and TEM (HF5000) from Hitachi Corporation in Tokyo, Japan to observe the changes in surface morphology in the slurry before and after the polishing process. The zeta potentials of manganese dioxide and alumina particles at different pH values (adjusted with NaOH or $HNO_{3}$ ) were determined at room temperature using a zeta plus particle apparatus (Nano-ZS, Malvern) from Malvern Instruments Ltd. in Malvern, UK. The laser particle size analyzer (Mastersizer 3000) from Malvern Instruments Ltd. in Malvern, UK was used to measure particle size distribution of powder in slurry before and after polishing. To detect changes in the chemical state before and after corrosion polishing, we used an XPS instrument referred to as the Thermo Scientific K-Alpha, with an excitation source of Al K α radiation (hv = 1486.6 eV).

## 3. Results and Discussion

### 3.1. Orthogonal Design Experiment

Material removal rate and surface roughness are two important parameters that can be used to evaluate the performance of CMP slurry. To achieve a high MRR and low surface roughness in the CMP of SiC crystal substrates, we selected three important factors of SiC CMP: pH value, oxidant content, and abrasive content. Five levels were selected for each factor. The experimental results are shown in Table 2.

We performed numerical calculations on the above results. Take factor pH, for example. The sums of the test objectives for factor pH are as follows. In the calculation results, $K_{jm}$ is the sum of the test results under factor *j* and level *m*; $k_{jm}$ is the average value of $K_{jm}$ at all levels; $R_{j}$ is the range in factor *j*.

$K_{pH=2}=0.52122+0.7665+0.79716+0.49056+0.64386=3.2193$ ,

$k_{pH=2}=3.2193/5=0.64386$ ,

$K_{pH=3}=0.55188+0.4599+0.70518+0.64386+0.64386=3.00468$ ,

$k_{pH=3}=3.00468/5=0.60094$ ,

$K_{pH=4}=0.49056+0.70518+0.52122+0.70518+0.73584=3.15798$ ,

$k_{pH=4}=3.15798/5=0.631596$ ,

$K_{pH=5}=0.52122+0.70518+0.70518+0.82782+0.79716=3.55656$ ,

$k_{pH=5}=3.55656/5=0.711312$ ,

$K_{pH=6}=0.58254+0.73584+0.67452+0.7665+0.70518=3.46458$ ,

$k_{pH=6}=3.46458/5=0.692916$ ,

$R_{pH}=0.711312-0.60094=0.110376.$

**Figure 1:**
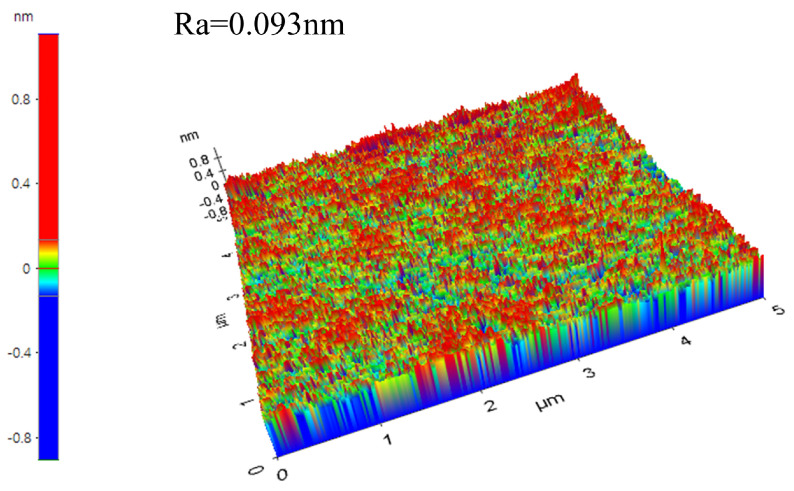
AFM image of SiC after polishing.

A similar process was used to calculate the K and k values of the other factors. The results are shown in more detail in Table 3:

The data listed in Table 3 show that the oxidant content has the greatest influence on MRR, while the influence of pH value is much smaller. The abrasive content has the smallest impact on MRR. According to the results of the orthogonal design experiment, the optimization combination is pH = 4, oxidant content = 5 wt%, and $Al_{2}O_{3}$ content = 5 wt%. Additionally, based on the basic slurry composition of the optimization combination, we tested the CMP SiC (0001) Si surface. The results shows that the MRR is 1.2 μm/h and the surface roughness Ra is 0.093 nm. Figure 1 represents the surface morphology of SiC after optimized slurry polishing indicate that a scratch-free and smooth substrate surface can be achieved after polishing by this slurry.

### 3.2. Analysis of Polishing Mechanism

To further investigate the role of aluminum oxide abrasive and its polishing mechanism in slurries with hypermanganate as the oxidizer, we utilized SEM, EDS, XPS, and zeta plus particle apparatus for analysis. During the experimental process, we selected a portion of the slurry that was thoroughly mixed before polishing and another portion of the slurry. For these two sets of slurries, we conducted centrifugation and drying processes to obtain powders.

**Figure 2:**
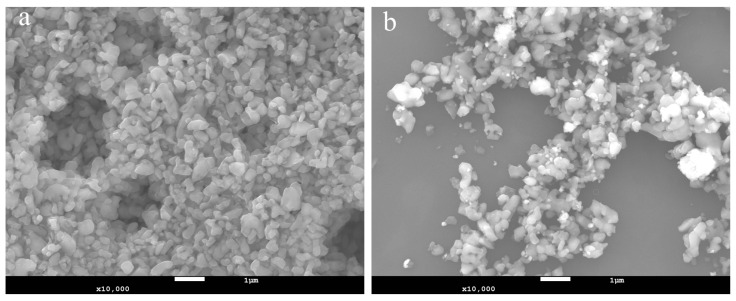
SEM diagram of the powder separated from the slurry (**a**) before and (**b**) after polishing.

Figure 2 presents SEM pictures of powders prepared from slurries before and after polishing at 10,000× magnification. The size of the powder particles before polishing is uniform, but after polishing, in addition to the normal-sized powder, small particles and agglomerated large particles could also be observed. The small particles may be alumina particles that broke during the polishing process, but we have yet to determine the composition of the large particle agglomerate.

**Figure 3:**
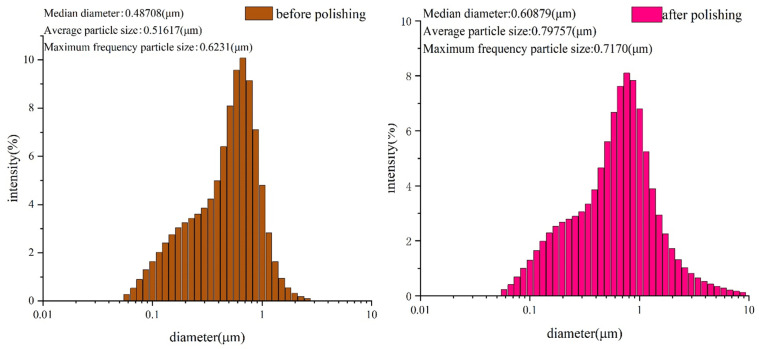
Particle size distribution of powder (**a**) before polishing and (**b**) after polishing.

Figure 3 shows the particle size distribution of the powder separated from the slurry before and after polishing. We selected alumina abrasive with a particle size of 500 nm. The figure shows that the powder separated from the slurry before polishing has a frequency diameter of 0.6231 μm. However, the average diameter and pitch diameter were still around 0.5 nm, indicating that a small portion of the abrasive was larger. After polishing, the average diameter of the powder reached 0.7978 μm and the frequency path was 0.717 μm. This indicates that the overall particle size of the abrasive changed significantly, causing the abrasive to aggregate.

**Figure 4:**
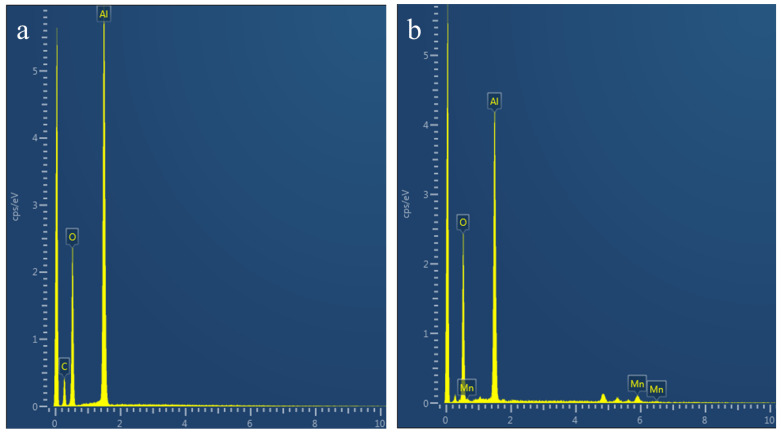
EDS energy spectrum of the powder separated from the slurry (**a**) before and (**b**) after polishing.

To clarify the composition of the agglomerated large particles, we used EDS to analyze the elemental composition and distribution of the powder before and after polishing. Figure 4 shows that, in the powder prepared with the slurry before polishing, except for the impurity element C, there are only O elements and Al elements in the element distribution diagram. In the powder prepared using the polished slurry, in addition to the C, O and Al impurities, Mn was also found.

**Figure 5:**
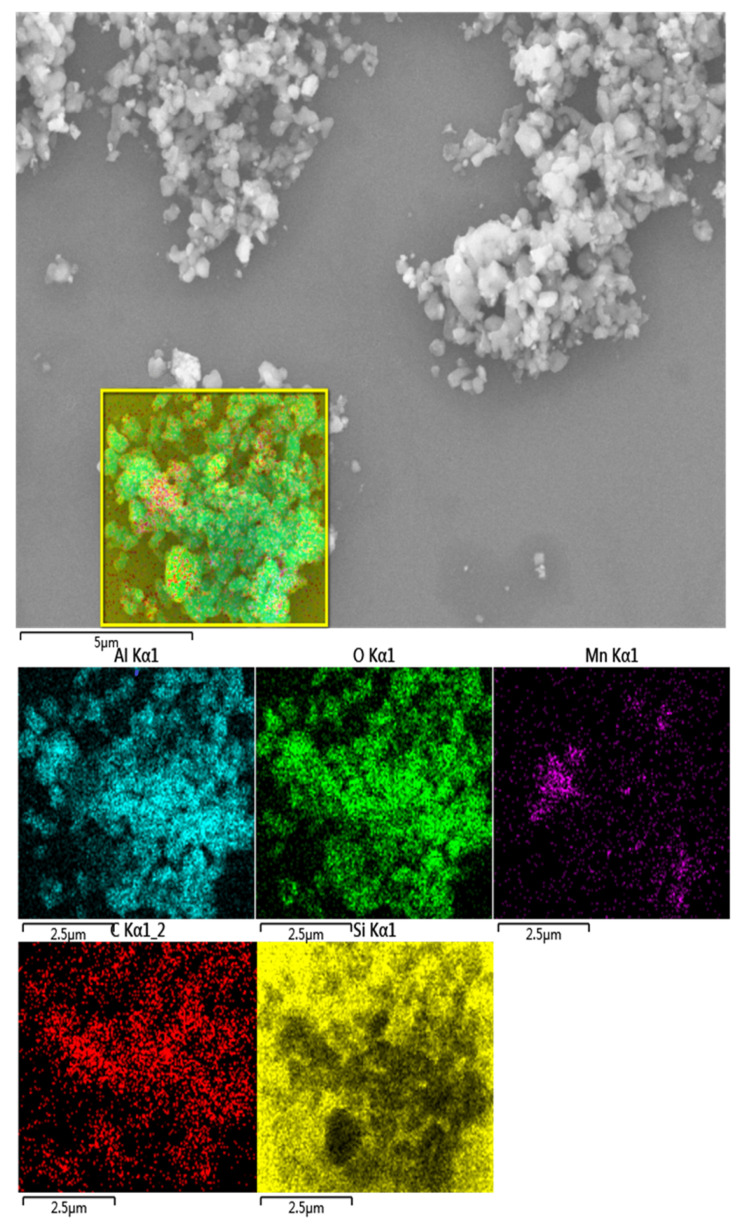
The image of distribution of elements in the powder after polishing using EDS.

We measured the elemental distribution of the agglomerated powder after polishing, finding a large number of Mn elements in which the powder was the agglomerate from Figure 5. Therefore, we speculate that the appearance of agglomerated particles may be related to the manganese element.

**Figure 6:**
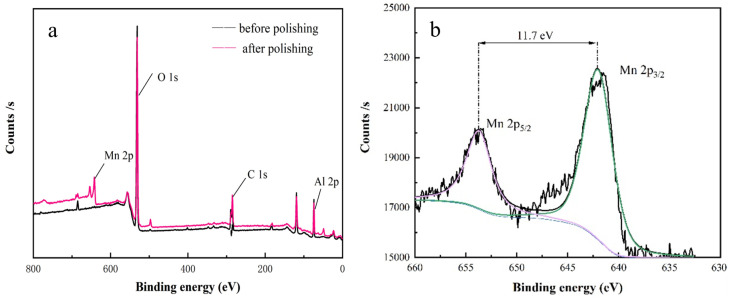
(**a**) Comparison chart of XPS full spectrum of slurry powder before and after polishing; (**b**) image of Mn 2p energy spectrum.

We subsequently conducted XPS analysis on both powders prepared from slurries before and after polishing. After charge correction, we used Origin for fitting and obtained the peak splitting information of the Mn 2p binding energy peaks. As shown in Figure 6a, the XPS image of the polished slurry powder contains more manganese than was present before polishing, which is consistent with the EDS results above. Figure 6b shows the Mn 2p core-level spectrum with two peaks located at 654.4 eV and 642.6 eV. This corresponds to the spin doublet of Mn $2p_{1/2}$ and Mn $2p_{3/2}$ with a separation of 11.7 eV, confirming the existence of $Mn^{4+}$ in the powder [14,15].

**Figure 7:**
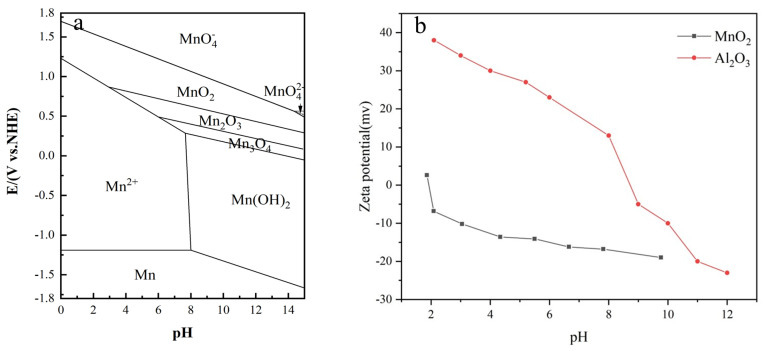
(**a**) Diagram of the relationship between pH and potential of Mn element. (**b**) Zeta potential plot of alumina and manganese oxide.

Figure 7a is the simple pH potential diagram of the Mn element [16], which shows that ${MnO_{4}}^{-}$ is reduced to $MnO_{2}$ at pH = 4. From Figure 7b, we can see that in the case of pH = 4, $MnO_{2}$ is negatively charged, $Al_{2}O_{3}$ is positively charged, and the two of them will attract each other under the action of static electricity, adsorbing together as a result. Therefore, we can hypothesize that the large particles in Figure 2b are caused by adsorption of $MnO_{2}$ and $Al_{2}O_{3}$ .

**Figure 8:**
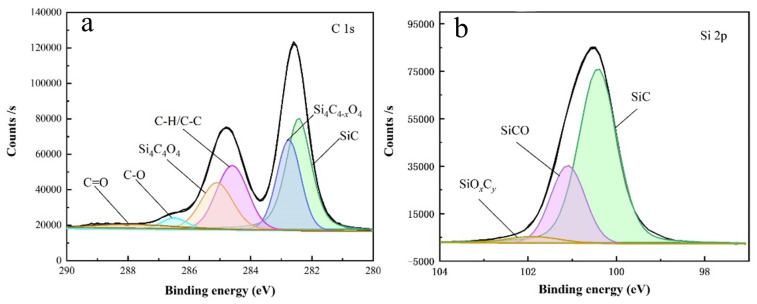
(**a**) C1s, (**b**) Si 2p energy spectrum of silicon carbide after polishing.

In order to further elucidate the polishing mechanism of silicon carbide, we analyzed the polished SiC surface using XPS. Figure 8 show the peak splitting diagrams of C1s and Si 2p for silicon carbide after polishing with slurry. By comparing these results with the XPS reference table and conducting an in-depth study of the literature, we successfully fitted the energy peaks of C 1s and Si 2p [17,18]. The C 1s binding energy peak contains six peaks: Si–C (282.4 eV), $Si_{4}C_{4-x}O_{4}$ (283.2 eV), C–C/C–H (284.6 eV), $Si_{4}C_{4}O_{4}$ (285.1 eV), C–O (286.1 eV), C=O (288 eV). The Si2p binding energy peak contains three main peaks: Si–C (100.4 eV), Si–C–O (101.1 eV), $SiO_{x}C_{y}$ (101.9 eV) [19]. Figure 7 shows that $MnO_{4}^{-}$ , as a strong oxidizing agent, can accept electrons, thus reducing it to a lower-valent manganese ion. In weakly acidic conditions, it is reduced to $MnO_{2}$ . The reaction equations are as follows:

(2) $$MnO_{4}^{-}+SiC\to MnO_{2}+Si_{x}C_{y}O_{z}$$

**Figure 9:**
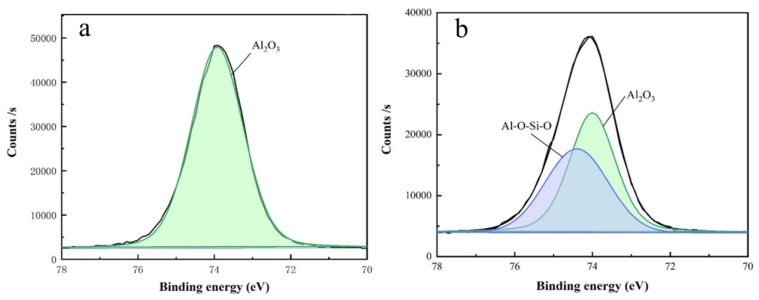
Al 2p binding energy peak splitting diagram of powder (**a**) before and (**b**) after polishing.

Figure 9 show the peak splitting of the Al 2p energy spectrum before and after polishing. Before polishing, Al 2p only has the peak of alumina. After polishing, because the alumina abrasive reacts with the oxide layer on the surface of silicon carbide, the structure of Al–O–Si–O is formed, and the manganese peak is not observed Al 2p, which indicates that $MnO_{2}$ in the slurry does not react with alumina, but is adsorbed on the surface of alumina.

**Figure 10:**
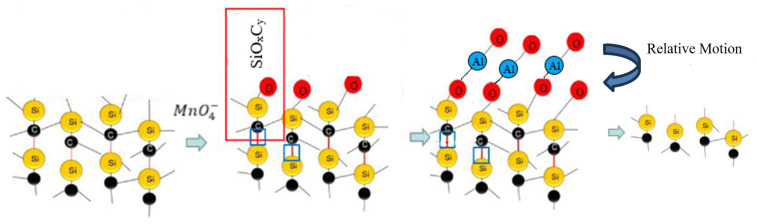
Schematic diagram of the polishing mechanism of the slurry $Al_{2}O_{3}$ .

We conclude that, during the polishing process, the surface of the silicon carbide is oxidized by hypermanganate in the polishing solution, forming an oxide layer. Under pressure and in an environment of relative motion, solutions that polish through abrasion rub the wafer surface. The contact point may produce an instantaneous high temperature, causing the alumina and oxide layer to adhere together and undergo a solid-state reaction [20,21,22]. The adhesion junction is sheared and slides under the action of friction, and the oxide layer is cut down and carried away by the flow of the slurry, successfully polishing the silicon carbide surface. At the same time, the hardness of manganese oxide adsorbed on the surface of alumina is relatively soft, which helps with the formation of a smooth surface with lower surface roughness. A schematic diagram of this process is shown in Figure 10.

## 4. Conclusions

In this article, we study the influencing factors of alumina slurry and analyze the mechanism of polishing silicon carbide using slurry. Our conclusions are as follows.

Firstly, we conducted 25 types of tests using different CMP slurries according to the orthogonal design table method. According to the range analysis method, the optimal CMP paste based on alumina ( $Al_{2}O_{3}$ ) abrasive for Si surface of SiC crystal substrate (0001) was obtained. According to the CMP test results of the optimized combination, the MRR is 1.2 μm/h and the surface roughness Ra is 0.093 nm.

Secondly, we found that during the polishing process, silicon carbide undergoes an oxidation reaction with hypermanganate in slurry, forming an oxide layer on the surface of silicon carbide and generating manganese dioxide. and due to the electrostatic effect of the two, $MnO_{2}$ will be adsorbed on the $Al_{2}O_{3}$ .

Lastly, we found that during the polishing process, the $Al_{2}O_{3}$ undergo a solid-state reaction with the oxide layer on the surface of silicon carbide. The oxide layer was cut off and carried away by the flow of slurry, successfully polishing the surface of silicon carbide.

## Figures and Tables

**Table 1:** Polishing parameters during the experiment.

Items	Value
Wafer	2-inch 4H-SiC
Polishing machine	Bruker CP-4
Polishing pad	Suba 800
Load (psi)	4
Polishing head rotation speed (r/min)	100
Polishing platen speed (r/min)	90
Polishing fluid flow rate (mL/min)	90
Polishing time (min)	60

**Table 2:** The experimental results of the orthogonal experiment.

Test Number	pH Value	Oxidant Content (wt%)	Abrasive Content (wt%)	MRR (μm/h)	Ra (nm)
1	2	1	1	0.5212	0.348
2	2	2	3	0.7665	1.277
3	2	3	5	0.7972	0.365
4	2	4	2	0.4906	0.122
5	2	5	4	0.6439	0.113
6	3	1	5	0.5519	0.248
7	3	2	2	0.4599	0.311
8	3	3	4	0.7052	0.355
9	3	4	1	0.6439	0.123
10	3	5	3	0.6439	1.358
11	4	1	4	0.4906	0.176
12	4	2	1	0.7052	0.809
13	4	3	3	0.5212	1.828
14	4	4	5	0.7052	0.129
15	4	5	2	0.7358	2.468
16	5	1	3	0.5212	0.225
17	5	2	5	0.7052	0.766
18	5	3	2	0.7052	0.408
19	5	4	4	0.8278	0.149
20	5	5	1	0.7972	1.970
21	6	1	2	0.5825	0.268
22	6	2	4	0.7358	0.264
23	6	3	1	0.6745	0.210
24	6	4	3	0.7665	0.110
25	6	5	5	0.7052	0.116

**Table 3:** The calculation results of parameters.

Parameter	pH Value	Oxidant Content (wt%)	Abrasive Content (wt%)
$K_{j1}$	3.2193	2.66742	3.34194
$K_{j2}$	3.00468	3.3726	2.97402
$K_{j3}$	3.15798	3.40326	3.2193
$K_{j4}$	3.55656	3.43392	3.40326
$K_{j5}$	3.46458	3.5259	3.46458
$k_{j1}$	0.64386	0.533484	0.668388
$k_{j2}$	0.60094	0.67452	0.594804
$k_{j3}$	0.631596	0.680652	0.64386
$k_{j4}$	0.711312	0.686784	0.680652
$k_{j5}$	0.692916	0.70518	0.692916
$R_{j}$	0.110376	0.171696	0.097812

## Data Availability

Data will be made available upon request.
