# Correction: Gong et al. Polishing Mechanism of CMP 4H-SiC Crystal Substrate (0001) Si Surface Based on an Alumina (*Al*_2_*O*_3_) Abrasive. *Materials* 2024, *17*, 679

**DOI:** 10.3390/ma17133295

**Published:** 2024-07-04

**Authors:** Juntao Gong, Weilei Wang, Weili Liu, Zhitang Song

**Affiliations:** 1 National Key Laboratory of Materials for Integrated Circuits, Shanghai Institute of Microsystem and Information Technology, Chinese Academy of Sciences, 865 Changning Road, Shanghai 200050, China; ztsong@mail.sim.ac.cn; 2 University of Chinese Academy of Sciences, No. 19, Yuquan Road, Shijingshan District, Beijing 100049, China; 3 Shanghai Xinanna Electronic Technology Co., Ltd., Shanghai 201506, China; awelly@mail.sim.ac.cn; 4 Zhejiang Xinchuangna Electronic Technology Co., Ltd., Haining 314406, China

In the original publication [1], there was an error regarding the affiliation for Juntao Gong. In addition to affiliation 1, the updated affiliations should include: “University of Chinese Academy of Sciences, No. 19, Yuquan Road, Shijingshan District, Beijing 100049, China”. The authors state that the scientific conclusions are unaffected. This correction was approved by the Academic Editor. The original publication has also been updated.
